# It Is Not Always Diverticular Bleeding: Fatal Subtle Primary Aorto-Duodenal Fistula Associated With Abdominal Aortic Aneurysm

**DOI:** 10.7759/cureus.34461

**Published:** 2023-01-31

**Authors:** Fouad Jaber, Saqr Alsakarneh, Kimberly Sanders, Ali Ibrahim, Hassan Ghoz, Wendell Clarkston, Charles McMahon

**Affiliations:** 1 Internal Medicine, University of Missouri-Kansas City, Kansas City, USA; 2 Gastroenterology and Hepatology, University of Missouri-Kansas City, Kansas City, USA; 3 Gastroenterology, University of Missouri-Kansas City, Kansas City, USA; 4 Gastroenterology and Hepatology, Mid-America Gastro-Intestinal Consultants, Kansas City, USA

**Keywords:** endovascular aortic repair (evar), gastrointestinal bleeding, abdominal aortic aneurysm, primary aorto-duodenal fistula, aorto-enteric fistula

## Abstract

Primary aorto-duodenal fistula (PADF) is a connection between the aorta and duodenum without prior aortic surgery. We present a case of an 80-year-old female who presented with hematochezia. She was vitally stable but later developed a large episode of hematemesis followed by cardiac arrest. A computed tomography angiogram (CTA) chest scan showed an abdominal aortic aneurysm (AAA) with no leakage or rupture. Esophagogastroduodenoscopy (EGD) demonstrated blood in the stomach and duodenum, but no source was identified. Tagged RBC scan showed massive hemorrhage in the stomach and proximal small bowel. Further review of the CT images identified a subtle PADF. The patient underwent endovascular aneurysm repair but died shortly after. Physicians should maintain a high awareness of PADF, particularly in elderly patients with obscure gastrointestinal bleeding with or without known AAA. Herald bleeding in the setting of an aortic aneurysm should raise suspicion for PADF even in the absence of extravasation on CTA.

## Introduction

Primary aorto-enteric fistula (PAEF) is a direct connection between a native aorta and the gastrointestinal tract (GI) without prior aortic surgery [1]. It is a rare entity with an incidence of 0.007 per million [1] and it accounts for less than 0.2% of all GI bleeding causes [2]. The classic triad of GI bleeding, abdominal pain, and pulsating abdominal mass [3] is only shown in 25% of PAEF [1,4]. We present a rare case of a subtle primary aorto-duodenal fistula (PADF) associated with abdominal aortic aneurysm (AAA) that resulted in fatal hemorrhagic shock.

## Case presentation

An 80-year-old female with a past medical history of AAA, peripheral arterial disease, persistent atrial fibrillation (on warfarin), and diabetes mellitus presented initially to an outside hospital with one episode of self-resolving hematochezia. Her vital signs were stable at that time, and her hemoglobin (Hgb) was 12 g/dL. Bleeding was attributed to diverticular bleeding, and she was discharged home with omeprazole. At home, the patient had an episode of hematemesis and became unresponsive. She underwent cardiopulmonary resuscitation (CPR) with a return of spontaneous circulation (ROSC). She was transported back to the outside hospital and emergently intubated on arrival. She was found to have a large amount of red blood from the mouth and rectum. The patient was hemodynamically unstable, with laboratory studies notable for Hgb of 8 g/dL, international normalized ratio (INR) 2.8, blood urea nitrogen (BUN) 26 mg/dL, and creatinine 1 mg/dL.

**Figure 1:**
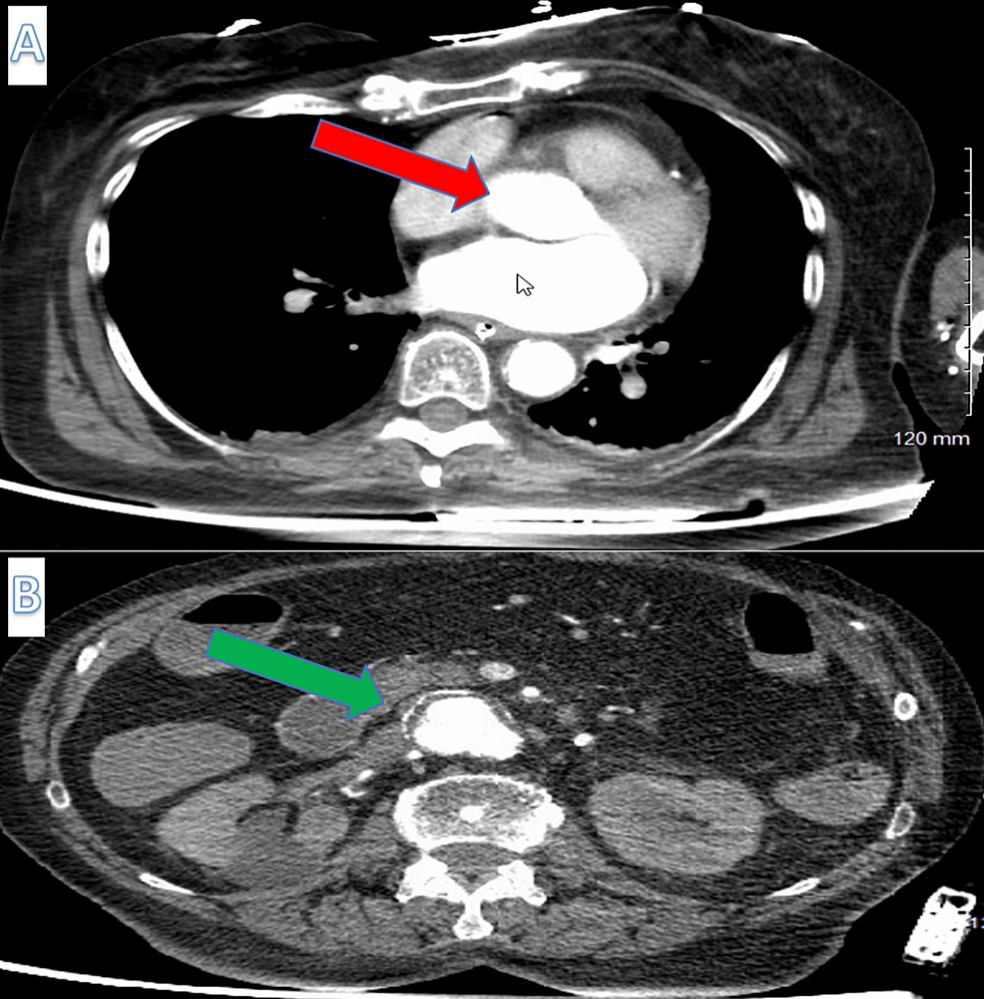
Computed tomography angiogram (CTA) chest and CTA abdomen and pelvis of the patient. The images show (A) CTA chest showing an unruptured 1.7 x 0.7 x 1.3 cm saccular aneurysm of the posterior wall of the ascending aorta (red arrow) and (B) CTA abdomen and pelvis showing diffuse aneurysmal dilation (green arrow) of infrarenal abdominal aorta measuring 4.4 x 4.3 cm with no leak or rupture along with a short segment of aortic dissection flap involving 4 cm long segment of aorta.

On admission to our facility, an orogastric tube was placed, and approximately 700 mL of bright red blood returned. Laboratory values were remarkable for Hgb of 6.2 g/dL and lactic acid of 6.9 mmol/L. She received four units of packed red cells, prothrombin complex concentrate (PCC), and vitamin K. Computed tomography angiogram (CTA) chest showed an unruptured 1.7 x 0.7 x 1.3 cm saccular aneurysm of the posterior wall of the ascending wall (Figure 1A). CTA abdomen and pelvis showed AAA (4.3 x 4.3 cm) with no leakage or rupture and no active bleeding detected (Figure 1B). Historically, a previous esophagogastroduodenoscopy (EGD) two years ago showed esophagitis and gastritis, with a biopsy negative for *Helicobacter pylori* (*H. pylori*). The patient was treated with a limited course of proton pump inhibitor therapy. The patient had no known history of GI bleeding, no non-steroidal anti-inflammatory drugs (NSAIDs) use, and no known liver disease. She drank one to two wine glasses daily.

**Figure 2:**
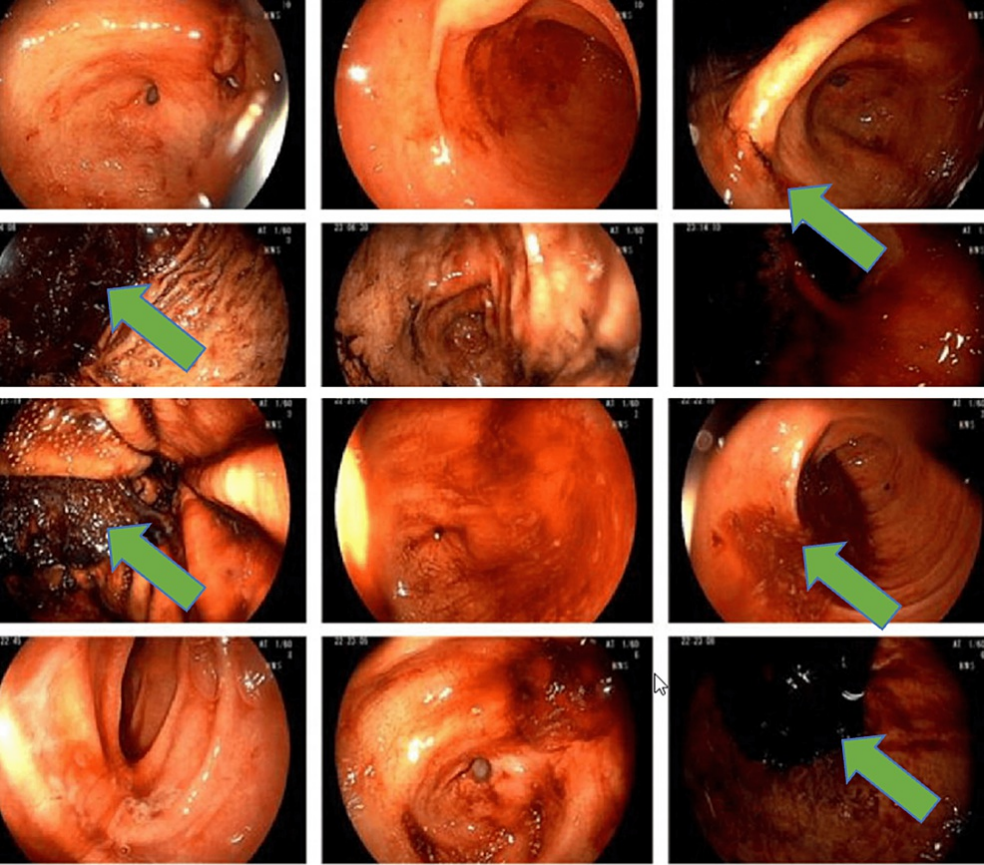
Esophagogastroduodenoscopy (EGD) showing clotted blood in the fundus, first and second parts of the duodenum (green arrows).

**Figure 3:**
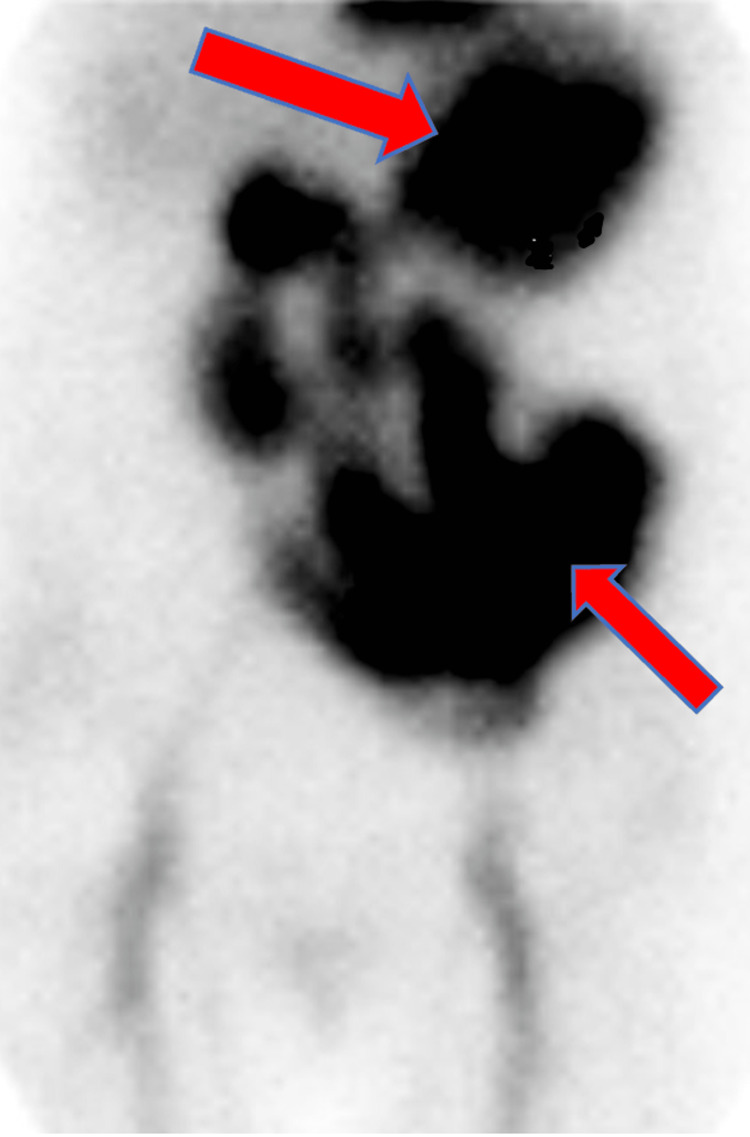
Tagged RBC scan showing massive blood (red arrows) throughout the stomach and proximal small bowel (red arrows).

Emergent EGD showed gastritis and clotted blood in the fundus as well as in the first and second parts of the duodenum (Figure 2). Multiple attempts to remove the clot with a snare and Roth net were unsuccessful. After extensive irrigation and suctioning, the underlying mucosa appeared normal without a definitive source of bleeding. However, refluxed blood was observed upon a second look. To localize the bleeding source, a tagged RBC scan was performed and showed active bleeding in the stomach and small bowel (Figure 3). She had another cardiac arrest, and ROSC was achieved. Multi-disciplinary teams discussion was ongoing. After further images review, expert radiologists identified a subtle aorto-enteric fistula that was felt to be the likely source of hemorrhagic shock.

The patient underwent endovascular aortic aneurysm repair (EVAR) with bifurcated endograft with plans to perform a stent graft explantation with aortic reconstruction later. The patient continued to deteriorate, and the hospital course was further complicated by a large right pneumothorax. She had continuous bloody drainage from the oropharynx and nasogastric tube, with hemoglobin trending down, requiring a massive blood transfusion protocol. CT head demonstrates significant cerebral swelling with early signs of herniation. After a discussion with the family, she was placed on comfort care, and then she expired soon after.

## Discussion

Aorto-enteric fistula (AEF), a direct connection between the aorta and the GI tract, is classified into primary and secondary types depending on the presence or absence of prior aortic surgery [1]. Secondary AEF is 10 times more common than primary AEF and usually results from erosion of the aortic prosthesis into the GI tract after previous aortic prosthesis reconstruction [5]. Most primary AEFs are caused by the expansion of the native aorta with mechanical compression of an aortic aneurysm against the GI tract, resulting in fibrotic and inflammatory destruction [2].

PADF is a connection between the infrarenal aorta and the duodenum and accounts for 80% of AEF [2,5]. The third part of the duodenum is involved in two-thirds of cases due to its fixed retroperitoneal location over the aorta [6]. Our patient was diagnosed with AAA measuring 3.8 cm in 2019. She most likely developed PADF secondary to continuous expansion with repeated trauma of AAA, 4.3 cm on recent CT, that resulted in the development of a fistula into the duodenum.

The clinical manifestation of PADF is characterized by herald GI bleeding followed by massive hemorrhage and exsanguination [1]. Herald bleeds are self-limiting and are reported in 30% of PADF [7]. It may be the only manifestation of fistulation and should raise suspicion of PADF [7]. The interval between herald bleeding and subsequent massive GI bleeding varies from hours to months [8]. However, in 50% of patients, the interval is around 24 hours [2], and in a third of patients, within 6 hours [8]. This is likely due to low blood pressure and thrombus formation obstructing the fistula and temporarily stopping bleeding [8]. Consistent with this, our patient had hematochezia with stable Hgb and vital signs 4 hours before readmission with massive hemorrhagic shock. We emphasize that detection of precursor bleeding in AAA may allow adequate time for diagnosis and treatment and potentially better outcomes. Our case is also an example of anchoring bias. The patient was initially discharged with diverticular hemorrhage as the most likely diagnosis. We emphasize that GI bleeding, especially in the elderly, may not always be attributed to the most common causes, such as diverticular bleeding. Even with stable vital signs and hemoglobin, hospitalization should be considered in such cases for further assessment and evaluation.

CTA of the aorta has 40-90% sensitivity and 33-100% specificity in detecting AEF [9]. PADF was not initially identified on the review of CTA abdomen and pelvis in our patient, which could be attributed to suboptimal contrast or lower sensitivity of CTA for detecting AEF. A careful review of radiographic images combined with a muti-team discussion led to the diagnosis.

The fact that the endoscopic detection rate for PADF is only 25% makes it particularly useful for ruling out other causes of upper GI bleeding [6,10]. This low detection rate may be due to EGD's limited visualization of the distal third of the duodenum. Hence, push enteroscopy should be considered in these patients. In our case, the EGD showed clotted blood in the stomach and proximal duodenum with unsuccessful clots removal. However, refluxed blood was observed upon second look, and this likely was from the distal duodenal fistula. To identify the source of the bleeding, tagged RBC scan was done and showed massive bleeding into the stomach and small intestine, raising the concern for PADF.

Treatment of PAEF includes reconstruction of the aorta and repair of the duodenum with an exploratory laparotomy [11]. Endovascular aortic repair (EVAR) can be used as a bridge approach in hemodynamically unstable patients [12]. The mortality rate in untreated cases is around 80-100%, while ranges from 30% to 56% in treated cases [8,13,14]. Our patient rapidly deteriorated and died shortly after EVAR.

## Conclusions

To conclude, physicians should maintain a high awareness index for PADF, especially for unknown etiology of upper GI bleeding in elderly patients with or without known AAA. Herald bleeding in the setting of an aortic aneurysm should raise suspicion for PADF even in the absence of extravasation on CTA. Finally, push enteroscopy should be considered to detect distal duodenal fistulas.
